# Effects of Agricultural Fungicide Use on Aspergillus fumigatus Abundance, Antifungal Susceptibility, and Population Structure

**DOI:** 10.1128/mBio.02213-20

**Published:** 2020-11-24

**Authors:** Amelia E. Barber, Jennifer Riedel, Tongta Sae-Ong, Kang Kang, Werner Brabetz, Gianni Panagiotou, Holger B. Deising, Oliver Kurzai

**Affiliations:** a Leibniz Institute of Natural Product Research and Infection Biology–Hans Knöll Institute, Jena, Germany; b Institute for Hygiene and Microbiology, University of Würzburg, Würzburg, Germany; c Institute for Agriculture and Nutritional Sciences, Martin Luther University Halle-Wittenberg, Halle (Saale), Germany; d Biotype GmbH, Dresden, Germany; e Department of Medicine, University of Hong Kong and State Key Laboratory of Pharmaceutical Biotechnology, University of Hong Kong, Hong Kong, China; University of Texas Health Science Center

**Keywords:** *Aspergillus fumigatus*, *Aspergillus*, antifungal resistance, azole, fungicide, population genomics, antibiotic resistance

## Abstract

Antibiotic resistance is an increasing threat to human health. In the case of Aspergillus fumigatus, which is both an environmental saprobe and an opportunistic human fungal pathogen, resistance is suggested to arise from fungicide use in agriculture, as the azoles used for plant protection share the same molecular target as the frontline antifungals used clinically. However, limiting azole fungicide use on crop fields to preserve their activity for clinical use could threaten the global food supply via a reduction in yield. In this study, we clarify the link between azole fungicide use on crop fields and resistance in a prototypical human pathogen through systematic soil sampling on farms in Germany and surveying fields before and after fungicide application. We observed a reduction in the abundance of A. fumigatus on fields following fungicide treatment in 2017, a finding that was not observed on an organic control field with only natural plant protection agents applied. However, this finding was less pronounced during our 2018 sampling, indicating that the impact of fungicides on A. fumigatus population size is variable and influenced by additional factors. The overall resistance frequency among agricultural isolates is low, with only 1 to 3% of isolates from 2016 to 2018 displaying resistance to medical azoles. Isolates collected after the growing season and azole exposure show a subtle but consistent decrease in susceptibility to medical and agricultural azoles. Whole-genome sequencing indicates that, despite the alterations in antifungal susceptibility, fungicide application does not significantly affect the population structure and genetic diversity of A. fumigatus in fields. Given the low observed resistance rate among agricultural isolates as well the lack of genomic impact following azole application, we do not find evidence that azole use on crops is significantly driving resistance in A. fumigatus in this context.

## Abstract (precis)

Antibiotic resistance is an increasing threat to human health. In the case of Aspergillus fumigatus, which is an environmental fungus that also causes life-threatening infections in humans, antimicrobial resistance is suggested to arise from fungicide use in agriculture, as the chemicals used for plant protection are almost identical to the antifungals used clinically. However, removing azole fungicides from crop fields threatens the global food supply via a reduction in yield. In this study, we survey crop fields before and after fungicide application. We find a low overall azole resistance rate among agricultural isolates, as well as a lack of genomic and population impact following fungicide application, leading us to conclude azole use on crops does not significantly contribute to resistance in A. fumigatus.

## INTRODUCTION

Aspergillus fumigatus is a globally distributed fungus responsible for an estimated 300,000 cases of invasive disease and more than 10 million cases of chronic and allergic disease globally each year (1). Humans inhale the infectious particles, or spores, on a daily basis but are actually an accidental host for the fungus, whose primary niche is soil and decaying vegetation. Management and prophylaxis against aspergillosis relies largely on the azole class of antifungals, with voriconazole and isavuconazole recommended as the first-line therapy (2). Unfortunately, clinical resistance to the azoles in A. fumigatus is an increasing problem, with some medical centers reporting rates as high as 30% in specific patient populations and similarly high rates for environmentally isolated A. fumigatus (3, 4). Regrettably, the mortality for resistant infections is upwards of 90% in some patient populations (5–7). While resistance can evolve during patient therapy (8, 9), the emergence of resistance in A. fumigatus has mainly been linked to the use of azoles in agriculture, as structurally similar and mechanistically indistinguishable compounds are heavily used for plant protection (10). This resistance has been described as collateral damage, as A. fumigatus is not a plant pathogen that is being directly targeted by fungicide treatments (11). The triazoles were first released for agriculture in 1973, well before they were first introduced to human medicine in the early 1990s, and are currently the most widely utilized antifungal compound group in agriculture due to their systemic distribution in treated plants, high efficiency, and broad spectrum of target pathogens (12, 13). Crops, particularly cereals and fruits, are sprayed multiple times each growing season at a recommended dose of 100 g/hectare to control powdery mildew, rust, septoria leaf blotch, and other phytopathogenic fungi (14). Currently, there are 32 azoles commercially available for plant protection (15) but only five in regular use in human medicine (16).

The most common azole resistance mechanism in A. fumigatus occurs via mutations in the target protein of azole fungicides, sterol 14α-demethylase (CYP51A, also called ERG11), a key enzyme of the ergosterol biosynthesis pathway. In A. fumigatus, the dominant resistance mechanism among both environmental and clinical isolates is a 34-bp tandem repeat (TR_34_) in the *cyp51a* promoter coupled with a leucine-to-histidine substitution (L98H) in the amino acid coding sequence, the net effect of which is an increase in gene expression as well as an alteration in both the stability of the target enzyme and the interaction between the protein heme cofactor of *cyp51a* and the azole ligand (17–19). Additional mutations that have been identified to confer azole resistance in A. fumigatus include other variations of the tandem repeat, such as TR_46_/Y121F/T289A and TR_53_, as well as other point mutations in the *cyp51a* coding sequence (20, 21).

Disease-causing fungi are responsible for roughly 20% of crop yield loss, with a further 10% loss postharvest (16), so the use of azoles is critical for securing the food supply. However, this must be balanced against the need to preserve the activity of the azoles for clinical use, and, as such, there is an urgent need to identify the contributions of azole fungicide use on food crops to the development of resistance in A. fumigatus. We address this through systematic soil sampling conducted on 10 agricultural sites in Germany over a 3-year period, including conventionally managed fields applying azoles fungicides as well as those practicing organic agriculture that do not use these compounds. In the largest published A. fumigatus sequencing effort to date, and the first to focus on the fungus in its natural niche, we also use whole-genome sequencing (WGS) to examine the impact of azole fungicides on the population genetics of 64 agriculturally isolated A. fumigatus isolates.

## RESULTS

### Variable abundance of Aspergillus fumigatus on agricultural sites in Germany.

**FIG 1:**
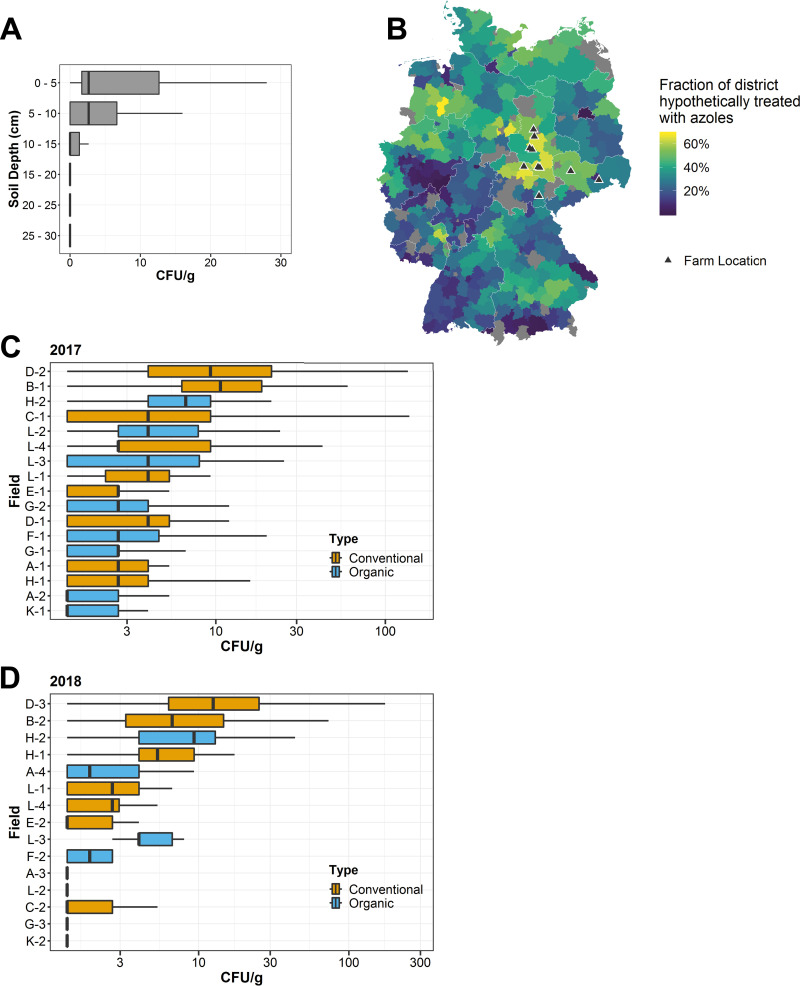
Abundance of A. fumigatus in the soil of conventional and organic farms. (A) A. fumigatus (CFU/g) at various soil depths. *n* = 10 samples per depth. (B) Estimated fungicide treatment rates and areas in Germany. The fraction of each district that is theoretically treated with fungicides was calculated using land use and organic agriculture share data reported by the Statistical Office of Germany in December 2016. Districts where no data on land use were available are shaded gray. (C and D) Abundance of A. fumigatus in the spring as measured by number of CFU/g soil in the spring of 2017 (C) and 2018 (D). For 2017, boxplots represent *n* = 50 soil samples per field for farms A, B, C, E, F, H, and K and *n* = 25 for farms D, G, and L. For 2018, *n* = 50 soil samples per field for farms A, B, C, D, E, G, H, and K and *n* = 25 for farms F and L. (E) Comparison of the mean number of CFU/g soil on farms between 2017 and 2018.

To examine the depth distribution of A. fumigatus in agricultural soils, we collected soil samples from a test field at 5-cm intervals down to a depth of 30 cm below the surface. A. fumigatus was most abundant in the top 5 cm of soil and was not significantly observed below a depth of 15 cm (Fig. 1A). As a result, only the top 5-cm layer of soil was collected in subsequent soil samples.

To identify whether our sampling areas are representative of azole and fungicide usage for Germany as a whole, we calculated the fraction of each administrative district that is theoretically treated with azoles in the context of agriculture using publicly available data. Overall, 51% of Germany is designated agricultural land (see Materials and Methods for data sources). However, not all this land is sprayed with azoles. For example, meadow or pastureland is rarely treated with fungicides. Additionally, approximately 7% of agriculture in Germany utilizes organic farming and does not apply azoles, with a range of 3 to 15% between the different federal states. Using land use data on arable farmland and permanent crop areas for each district in Germany, we calculated that the mean fraction of potentially azole-treated area in Germany is 32% (for the districts where data are available), with a range of 1 to 68% (Fig. 1B). The districts where we performed our soil sampling were almost all above the average for Germany, with a mean of 52% potentially azole-treated hectares and a range of 30 to 65%.

To examine the inter- and intrafield variability in the density of A. fumigatus on agricultural fields, soil samples were taken from nine conventional and eight organic fields before the growing season in 2017. Conventional fields were also sampled again after the vegetative period and application of azoles. In total, 2,875 soil samples were taken between 2016 and 2018 (see Table S1 in the supplemental material). The predominant crops being grown were cereals such as wheat and barley, but several apple orchards were also sampled. Of the fields sampled during this period in 2017, 67% of the soil samples taken were positive for A. fumigatus, with a large range between fields (28 to 100%) (Fig. 1C). We also observed a large degree of variation in the mean number of CFU per gram of soil between different fields, with some fields having 30× higher A. fumigatus density over others (0.7 to 18.8 CFU/g).

10.1128/mBio.02213-20.4 TABLE S1 Summary of the agricultural sites surveyed, 2016 to 2018. Download Table S1, DOCX file, 0.02 MB. Copyright © 2020 Barber et al. 2020 Barber et al. This content is distributed under the terms of the Creative Commons Attribution 4.0 International license.

To examine the stability of A. fumigatus population sizes in agricultural soil, we investigated the same farms a year later in the spring of 2018 and repeated the soil sampling on eight conventional and seven organic fields. Due to crop rotation, it was not possible to sample the same fields as the previous year, except for the apple orchards on farms H and L. During the 2018 sampling period, an overall lower proportion of samples were positive for A. fumigatus (51% with a range of 10 to 96% between different fields) (Fig. 1D), but the mean number of CFU per gram for the 1,000 samples was similar to that of the previous year (5.18 CFU/g soil for 2017 compared to 5.74 CFU/g for 2018). As in the previous year, there was a large variability in the mean number of CFU of A. fumigatus present between fields. When comparing the six apple fields that were sampled over consecutive years, we did not observe a consistent trend in the stability of A. fumigatus population size. Two fields showed similar levels of A. fumigatus between 2017 and 2018, while two fields showed an increased abundance between the years, and the remaining two fields showed a significant reduction in abundance (Fig. S1A). To investigate potential factors that might support a higher abundance of A. fumigatus, we compared the total organic carbon (TOC) content of a random selection of samples with the number of CFU per gram for A. fumigatus, but we did not detect a clear relationship between the two (Fig. S1B). Altogether, we observed a nonuniform distribution of A. fumigatus in soil samples taken from the same field and a large degree of heterogeneity between fields.

10.1128/mBio.02213-20.1 FIG S1 A. fumigatus abundance does not correlate with total organic carbon content of soil. *n* = 170 soil samples (10 per field from 17 fields on 10 different farms). Download FIG S1, EPS file, 1.0 MB. Copyright © 2020 Barber et al. 2020 Barber et al. This content is distributed under the terms of the Creative Commons Attribution 4.0 International license.

### Variable effects of fungicide application on A. fumigatus abundance.

To examine the impact of fungicide application and the azoles on A. fumigatus in agricultural soil, we performed additional soil sampling on the conventional fields surveyed in the spring at the end of the vegetative period and after several months of fungicidal crop protection. A schematic illustration of the soil sampling and fungicide application timelines for 2017 and 2018 can be found in Fig. S2A and B. Unfortunately, the fungicide history for farms H and L was not available to us. Fields were typically treated with fungicides twice during the growing period, and azoles were by far the most dominant class of fungicide applied. Every application recorded contained at least one azole. However, fungicides are often applied as commercially available cocktails of different chemicals, so other classes were also present in 0 to 55% of applications in 2017 and 2018 (summarized in Fig. S2C).

10.1128/mBio.02213-20.2 FIG S2 Fungicide history and schematic representation of the sampling time points and fungicide applications for the fields analyzed in this study. (A and B) Schematic representation of the sampling time points and fungicide applications for the fields sampled in 2017 (A) and 2018 (B). Soil sampling time points are marked in dark grey, while fungicide applications are noted in a combination of red and light grey, indicating the relative proportion of azoles within the fungicide cocktail applied. (C) Overall frequency of fungicide classifications applied to the fields sampled during 2017 and 2018. Fungicides are categorized by their Fungicide Resistance Action Committee (FRAC) mechanism of action, and a frequency of 100% means that a category was present in every fungicide application on every field analyzed that year. Download FIG S2, EPS file, 1.1 MB. Copyright © 2020 Barber et al. 2020 Barber et al. This content is distributed under the terms of the Creative Commons Attribution 4.0 International license.

**FIG 2:**
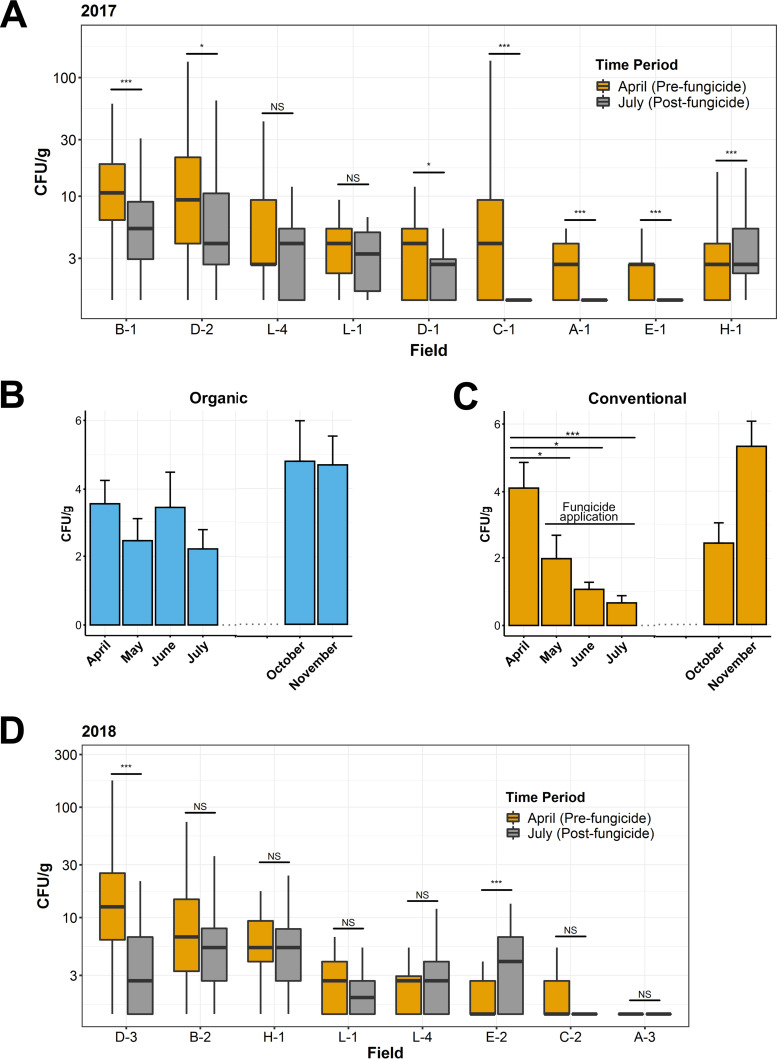
Abundance of A. fumigatus in the soil of conventional farms before and after the vegetative period and fungicide application. (A and D) A. fumigatus (CFU/g) on conventional fields sampled in April, prior to the vegetative period and fungicide application (orange), and in July, after the vegetative period and 3 months of fungicide application, including azole fungicides (gray) in 2017 (A) and 2018 (D). *, *P ≤ *0.05; **, *P ≤ *0.01; ***, *P ≤ *0.001; NS, not significant; determined by Mann-Whitney U test. (A) Boxplots represent *n* = 50 samples per field and time point for farms A, B, C, E, and H and *n* = 25 samples for farms D and L. (D) *n* = 50 samples per field and time point for farms A, B, C, D, E, and H and *n* = 25 samples for farm L. (B and C) A. fumigatus (CFU/g) during the months of April, May, June, July, October, and November of a conventional field applying fungicides from May to July (C) and an organic field not applying nonnatural fungicides (B). Bars represent means ± standard errors of the means from 50 soil samples per month. No significant difference was found in abundance between the months of April, May, June, and July for the organic field using a Kruskal-Wallis test. In contrast, we found a significant difference (*P = *0.004) for the abundances in this time period on the conventional field. *P* values from subsequent pairwise comparisons between months are indicated. *, *P ≤ *0.05; **, *P ≤ *0.01; ***, *P ≤ *0.001; determined by Wilcoxon signed rank.

When comparing the amount of A. fumigatus on fields before fungicide application to that after fungicide application and azole exposure, we detected a significant reduction in the number of CFU per gram of soil on the majority of fields in 2017 (Fig. 2A), even though it is not being directly targeted as a plant pathogen. To investigate whether this reduction in agricultural A. fumigatus populations was the result of fungicide application and not a seasonal effect from comparing April to July, monthly soil samples were taken from a conventional field and an organic field not treated with azoles or other nonnatural fungicides as a control. Samples were taken beginning in April, before azoles were applied to the conventional field, through the harvest period in July, and then additional samples were taken in October and November to allow for a period without fungicide application. From April to July, the conventional field was sprayed with azoles every 3 to 5 weeks. When analyzing the abundance of A. fumigatus on the organic field, we did not observe any significant differences between the abundance recorded monthly between April and July (Fig. 2B). However, the conventional field showed a significant reduction in abundance between April and May, corresponding to the beginning of the azole application period, and this reduction was maintained through the rest of the azole application period (Fig. 2C).

When comparing A. fumigatus density before and after fungicide treatment in 2018, we did not observe the same reduction in abundance, and most fields did not show significant changes between the time points (Fig. 2D). In fact, only one of eight fields sampled showed a statistically significant reduction in A. fumigatus abundance. Altogether, the impact of fungicide application on A. fumigatus abundance was variable between fields and more so between years, as other environmental factors also appear to influence A. fumigatus population size in agricultural soil.

### Reduced susceptibility to agricultural azoles in populations isolated after the growing season and azole exposure.

**FIG 3:**
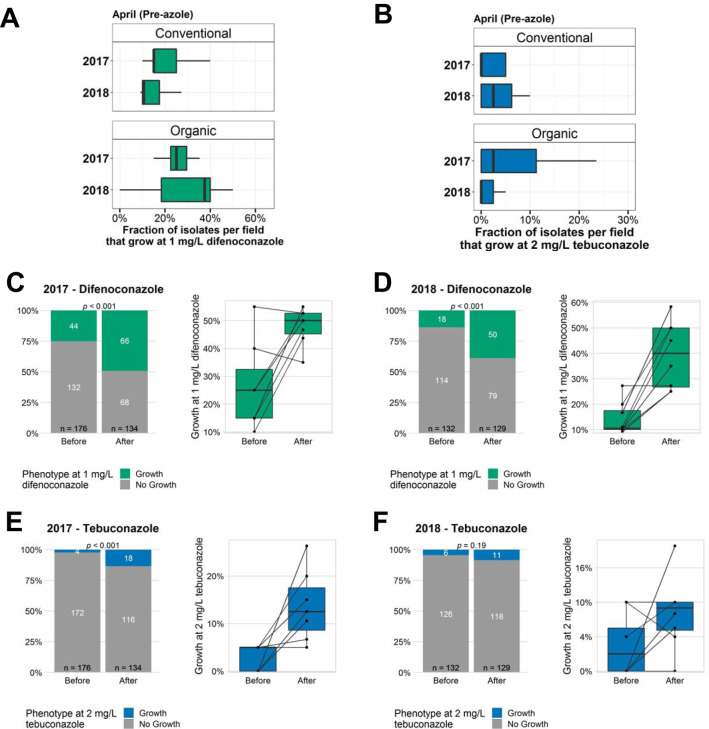

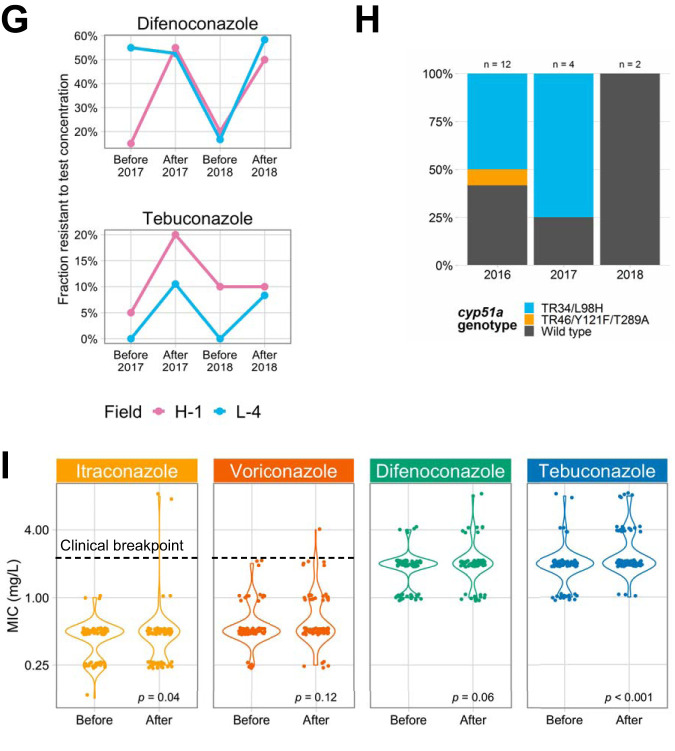
Azole resistance among agricultural A. fumigatus. (A and B) Fraction of the isolates per field that grow at 1 mg/liter difenoconazole (A) and 2 mg/liter tebuconazole (B). For 2017, *n* = 340 isolates from 9 conventional and 8 organic fields, ≈20 isolates per field, were used. For 2018, *n* = 213 isolates from 8 conventional and 7 organic fields, ≈20 isolates per field, were used (full summary in Tables S2 and S3). (C to F) Comparison of the proportion of isolates that grow at 1 mg/liter difenoconazole (C and D) and 2 mg/liter tebuconazole (E and F) before and after the vegetative period and fungicide application. For 2017 (C and E), *n* = 275 isolates from 7 fields were used, and for 2018, *n* = 261 isolates from 8 fields were used (full summary in Tables S4 and S5). (Left) Overall summary of all isolates tested that year. (Right) Within-field changes (*n* ≈ 40 isolates per field; 20 before, 20 after). *P* values were calculated by Wilcoxon signed-rank test between before and after values. (G) Temporal changes in antifungal susceptibility of A. fumigatus on apple fields sampled before and after fungicide exposure over a 2-year period. Shown is the fraction of isolates that can grow at 1 mg/liter difenoconazole (top) and 2 mg/liter tebuconazole (bottom). *n* = 12 to 20 isolates/field and time point. (H) *cyp51a* genotypes of isolates resistant to one or more medical azole. (I) MICs of agricultural A. fumigatus isolated before and after azole exposure. *n* = 159 randomly selected isolates from 2017 and 2018; *n* = 80 before and *n* = 79 after. *P* values were calculated by Wilcoxon signed rank test between before and after values.

To assess the susceptibility of A. fumigatus to commonly applied agricultural azoles, we screened 435 isolates from 2017 and 342 isolates from 2018 for their ability to grow at a set concentration of difenoconazole and tebuconazole (approximately 20 isolates per field and sampling point). To limit potentially clonal isolates from skewing the results, a maximum of two isolates per soil sample were included for testing. As there are no established breakpoints for defining resistance to these compounds in A. fumigatus, we selected concentrations that mimicked MIC_90_ values for these azoles (1 mg/liter for difenoconazole and 2 mg/liter for tebuconazole). When examining conventional and organic farms in the spring, we observed a wide range in the fraction of isolates per field that were able to grow when challenged with agricultural azoles. For difenoconazole, this ranged from 10 to 55% per field in 2017 (*n* = 17 fields, 320 isolates in total) and 0 to 50% in 2018 (*n* = 15 fields, 261 isolates in total) (Fig. 3A and Table S2). For tebuconazole, the rates ranged from 0 to 25% in 2017 and 0 to 20% in 2018 (Fig. 3B and Table S2). We did not detect any significant differences in the rates between conventional and organic fields in the spring or between fields growing different crops (cereals or apples).

10.1128/mBio.02213-20.5 TABLE S2 Resistance summary of the fields sampled during the spring of 2017 (A) and 2018 (B). Download Table S2, DOCX file, 0.02 MB. Copyright © 2020 Barber et al. 2020 Barber et al. This content is distributed under the terms of the Creative Commons Attribution 4.0 International license.

10.1128/mBio.02213-20.6 TABLE S3 Resistance summary of the fields sampled before and after the vegetative period and azole application in 2017 (A) and 2018 (B). Download Table S3, DOCX file, 0.02 MB. Copyright © 2020 Barber et al. 2020 Barber et al. This content is distributed under the terms of the Creative Commons Attribution 4.0 International license.

10.1128/mBio.02213-20.7 TABLE S4 MICs for medical and agricultural azoles in isolates displaying resistance to at least one medical azole. Download Table S4, DOCX file, 0.02 MB. Copyright © 2020 Barber et al. 2020 Barber et al. This content is distributed under the terms of the Creative Commons Attribution 4.0 International license.

10.1128/mBio.02213-20.8 TABLE S5 Primers used for amplifying and sequencing *cyp51a*. Download Table S5, DOCX file, 0.01 MB. Copyright © 2020 Barber et al. 2020 Barber et al. This content is distributed under the terms of the Creative Commons Attribution 4.0 International license.

Given the reduction in the A. fumigatus population size observed on most conventional fields following fungicide application in 2017 and more variably on fields in 2018, we wanted to examine the effect of fungicides on the local azole susceptibility following the vegetative period and several months of fungicide application. We observed an increase in the proportion of isolates that were able to grow at the test concentrations of difenoconazole (1 mg/liter) and tebuconazole (2 mg/liter) for fields sampled after azole exposure compared to the same field in the spring prior to azole application (Fig. 3C and D and Table S3). We detected a 1.97-fold increase in the proportion of isolates that were resistant to our test concentration of difenoconazole in 2017, with a range from −1.1- to 4.5-fold for individual fields (Fig. 3C). In 2018, this increased to 2.84-fold, with a range of 1- to 4.5-fold increase for individual fields (Fig. 3D). We also saw a similar increase in the fraction of isolates with reduced susceptibility to our test concentration of tebuconazole after azole exposure compared to that before. In 2017, we observed a 5.9-fold increase in the number of isolates that were able to grow at the test concentration after azole exposure versus before exposure, with a range of 0 to 25.0% for individual fields (Fig. 3E). In 2018, we detected a more modest 1.9-fold, with a range of −5 to 18.2% for individual fields (Fig. 3F).

For the fields growing cereals, we were not able to sample the same fields over subsequent years due to crop rotation and the fields not being in use the following year. However, we were able to compare the same apple fields in both 2017 and 2018. We tracked the local susceptibility to agricultural azoles in these fields over two consecutive years at two time points, in the spring prior to fungicide application and just prior to harvest after ≈3 months of fungicide application. The fraction of isolates that were able to grow in the presence of 1 mg/liter difenoconazole or 2 mg/liter tebuconazole was, in general, low in the spring and increased after fungicide application (Fig. 3G and Table S3). Interestingly, in the spring of 2018, the proportion had returned to a level comparable to what we observed in the spring of 2017, indicating that the reduced susceptibility is transient and recedes when the selective pressure imposed by fungicide is removed. In summary, we found a wide range of susceptibilities to agricultural azoles between different fields but a consistent decrease in susceptibility following the growing season, fungicide application, and azole exposure. However, this change is seemingly transient or reversible, and the A. fumigatus populations from fungicide-treated fields typically returned to what they were prior to fungicide application by the following spring.

### Resistance to medical azoles in agricultural A. fumigatus isolates.

We next examined our isolate collection for resistance to medical azoles and determined the proportion that would be considered clinically resistant. Using the VIPcheck agar-based screening method, followed by broth microdilution for isolates showing growth on agar-containing wells (22, 23), we determined that only a very small fraction of A. fumigatus organisms isolated from agriculture showed resistance to itraconazole, voriconazole, or posaconazole in 2016 to 2018 (Table 1). The overall resistance rate to itraconazole among all isolates collected was higher than that for other compounds, with 3.0% (11/333) of isolates being resistant in 2016, 0.7% in 2017 (4/460), and 0.6% (2/322) in 2018. We observed lower resistance rates for posaconazole and voriconazole, with only 2.1% (7/333) being resistant in 2016, 0.7% (4/460) in 2017, and 0.0% (0/322) in 2018. As there are no clinical breakpoints established for agricultural azoles, we calculated epidemiological cutoff values (ECOFFs) for difenoconazole and tebuconazole using MICs from 160 randomly selected isolates from 2017 and 2018. Using these values, we found that 1.3% of the 160 isolates had MICs above the ECOFF for difenoconazole (2 mg/liter), and 4.4% of isolates had MICs above the ECOFF for tebuconazole (2 mg/liter) (Table 2). As has been described previously (24), isolates resistant to one or more medical azoles often displayed elevated MICs to agricultural azoles, indicating cross-resistance (Table S4).

**TABLE 1:** Azole resistance rates among agriculturally isolated A. fumigatus isolates^*a*^

Year	*n*	Resistance rate (%)		
		ITR	POS	VOR
2016	333	3.0	2.1	2.1
2017	460	0.7	0.7	0.7
2018	322	0.6	0	0

a Isolates were screened for potential azole resistance to itraconazole (ITR), posaconazole (POS), and voriconazole (VOR) using VIPcheck agar-based screening. Resistance was confirmed and MICs determined via EUCAST broth microdilution testing.

**TABLE 2:** MIC_50_ for medical and agricultural azoles calculated from 160 randomly selected isolates as well as ECOFF_95_ and the fraction of isolates with MICs above this value

Azole	MIC_50_^*a*^	ECOFF^*b*^	Fraction of isolates above ECOFF (%)
Itraconazole	0.5	2	1.3
Voriconazole	0.5	2	0.6
Difenoconazole	2	8	1.3
Tebuconazole	2	8	4.4

a Calculated from 160 randomly selected isolates.

b Rounded up to next dilution.

To quantify what mutations were responsible for azole resistance in the population analyzed, we genotyped the *cyp51a* locus encoding the azole target enzyme for all isolates resistant to one more medical azoles using Sanger sequencing (*n* = 18). We found that the most dominant DNA alteration observed was the well-characterized TR_34_/L98H mutation (Fig. 3H and Table S4). This *cyp51a* genotype accounted for 6/12 (50%) of resistant isolates in 2016 and 3/4 (75%) in 2017. In 2018, we identified only two resistant isolates among the 322 screened, and both had wild-type *cyp51a* loci. However, both of these isolates were only weakly resistant to itraconazole, but not other azoles, with itraconazole MIC values right at the breakpoint of 2 to 4 mg/liter. For comparison, resistant isolates from other years had MIC values of >8 mg/liter. In total, the genetic cause of resistance remained unknown for 8 isolates from 2016 to 2018.

Since the majority of fields had no resistant isolates, it was not possible to effectively compare resistance rates among agricultural A. fumigatus organisms for medical azoles before and after fungicide treatment. In lieu of this, we examined the MIC distribution for isolates collected before and after the growing season and fungicide application. Examining 79 isolates from the before period and 79 isolates from after azole exposure, we observed a shift in the MIC distribution toward higher MICs for all azoles examined, both medical and agricultural (Fig. 3I). However, the median MICs remained unchanged, indicating that the majority of the population following azole exposure does not exhibit a change in MIC. Taken together, these results indicate that the rate of resistance to clinical azoles among environmental A. fumigatus organisms isolated from agricultural environments is low overall and that exposure to agricultural azoles alone or in combination with other fungicides causes a minor increase in MIC values for some of the population, but the majority of isolates are left unchanged.

### No distinct population structure for A. fumigatus isolates from different sampling sites.

To better understand the population structure of A. fumigatus in the environment and how it is impacted by the azoles, we performed whole-genome sequencing on isolates from four conventional farms collected before and after azole exposure. Sixty-four isolates were sequenced by Illumina paired-end sequencing, representing eight isolates per farm and time point. Isolates from each field and time point were randomly selected, with a maximum of two per soil sample to avoid sequencing of clonal isolates. Raw reads were checked for quality and then aligned to the Af293 reference genome, resulting in a median depth of coverage after mapping of 31.5× (range, 10× to 90×) and a median genome coverage median of 94% (range, 92.8 to 96.7%) (Data Set S1). Consistent with what has been observed among sequenced clinical strains (25), isolates differed from the Af293 reference by a median of 84,690 single-nucleotide variants (SNVs) or 2.88 SNVs/kb, with a range of 65,854 to 146,055 SNVs. The analysis of copy number variations (CNVs) identified 8,277 unique CNVs in total, with a median of 3,115 CNVs per isolate (range, 1,666 to 4,532 CNVs). These CNVs were further delimited into a median of 2,589 deletions (range, 1,247 to 4,405) and 5,687 insertions (range, 3,872 to 7,030).

10.1128/mBio.02213-20.10 DATA SET S1 Read counts, mapping, and genome coverage statistics for the WGS samples. Download Data Set S1, XLSX file, 0.02 MB. Copyright © 2020 Barber et al. 2020 Barber et al. This content is distributed under the terms of the Creative Commons Attribution 4.0 International license.

**FIG 4:**
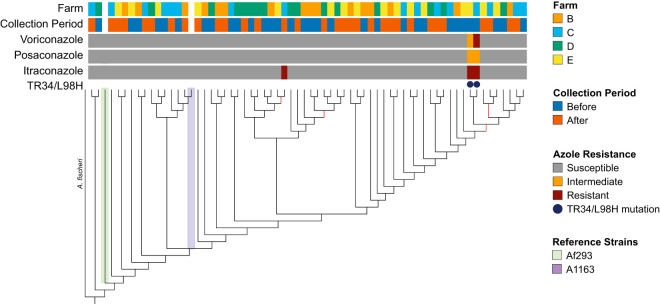
Phylogeny of agricultural A. fumigatus isolates from before and after the vegetative period and azole application. From top to bottom, the colored bars indicate the farm where the isolate was collected, the collection period, voriconazole resistance (susceptible, intermediate, or resistant, according to EUCAST definitions), posaconazole resistance, itraconazole resistance, and the presence of the TR_34_/L98H allele in *cyp51a*. A. fischeri is indicated as an outgroup, and the two A. fumigatus reference strains, Af293 and A1163 (CEA10), are also marked. Branches with support values of less than 0.9 are marked in red.

A maximum likelihood phylogeny based on SNVs indicated no population stratification among isolates from different farms and regions of Germany (Fig. 4). To more directly assess the association between genetic and geographic distance, we performed a Mantel test correlating a geographic distance matrix with the fixation index (F_ST_) genetic distance matrix and observed no significant association between the two. When considering the azole-resistance status of the isolates, the two itraconazole-resistant TR_34_/L98H isolates clustered next to each other, despite originating from separate farms, while the third itraconazole-resistant isolate with an undefined resistance mechanism was on a distinct branch. Despite being the nearest sequenced neighbors, the two TR_34_/L98H isolates were genetically distinct, each possessing 19,439 and 60,841 unique SNVs not shared by the other isolate, along with 67,196 common SNVs relative to Af293.

Comparative analysis of molecular variance (AMOVA) indicated that the majority of the variation seen among the 64 isolates came from the population as a whole (within sample) (94.8%) and between samples (5.0%) (Table 3). There was no significant molecular variance between farms (0.2%), with the exception of modest variation between farm B and farm C (1.2% of variation observed). Weighted Weir and Cockerham’s fixation indexes (F_ST_) for each farm were essentially zero, indicating an interbreeding, panmimetic population with no separation between farms (Fig. S3A). Analysis of copy number variation (V_ST_), estimating population differentiation based on copy number variation, also indicated no subdivision among the farms (Fig. S3B). To examine the genetic diversity within farms, nucleotide diversity (the average number of nucleotide differences per site for all possible pairs in the population or *π*) and the number of polymorphic sites (Watterson estimator, or θ) were calculated along 5-kb windows with a 500-bp step size for each farm population. Farm C showed the greatest intrafarm diversity, while farm E showed the smallest (Fig. S3C and D). Taken together, there was no population differentiation between A. fumigatus isolates from different farms in Germany, a finding in line with the fungus’ capacity for aerosol dispersal.

**TABLE 3:** AMOVA between farms^*a*^

Farm	Total variation	Variation within samples (%)	*P* value	Variation between samples (%)	*P* value	Variation between farms (%)	*P* value
All farms	289,735.7	274,622.7 (94.8)	<0.001	14,629 (5)	<0.001	484 (0.2)	0.303
B vs C	147,358.0	139,257.7 (94.5)	<0.001	6,386.2 (4.3)	<0.001	1,714.1 (1.2)	0.028
B vs D	190,425.2	181,470 (95.3)	<0.001	9,002.4 (4.7)	<0.001	−47.2 (0.0)	0.429
B vs E	162,874.7	155,745.5 (95.6)	<0.001	6,879.3 (4.2)	<0.001	249.9 (0.2)	0.211
C vs D	130,625.7	122,692.8 (93.9)	<0.001	7,932 (6.1)	<0.001	1 (0.0)	0.355
C vs E	105,529.1	99,521.4 (94.3)	<0.001	6,098.3 (5.8)	<0.001	−90.6 (−0.1)	0.414
D vs E	143,684.1	135,847.0 (94.5)	<0.001	8,382.7 (5.8)	<0.001	−545.6 (0.4)	0.890

a Calculated using isolates from before and after azole application. *n* = 16 isolates per farm.

10.1128/mBio.02213-20.3 FIG S3 Population genetic comparisons between farms using isolates from before and after azole exposure. *n* = 16 isolates per farm. (A and B) Heat map representing weighted Weir and Cockerham F_ST_ values (A) and V_ST_ values (B) between farms. Blue colors represent more similarity between populations, while red represents greater population differentiation. (C and D) Comparison of nucleotide diversity (π) (C) and nucleotide polymorphism (Watterson estimator, or θ) (D) between farms. Values were calculated from 5-kb sliding windows with a 500-bp step size. Download FIG S3, EPS file, 1.4 MB. Copyright © 2020 Barber et al. 2020 Barber et al. This content is distributed under the terms of the Creative Commons Attribution 4.0 International license.

### Changes in population genetics following azole exposure vary by field.

**FIG 5:**
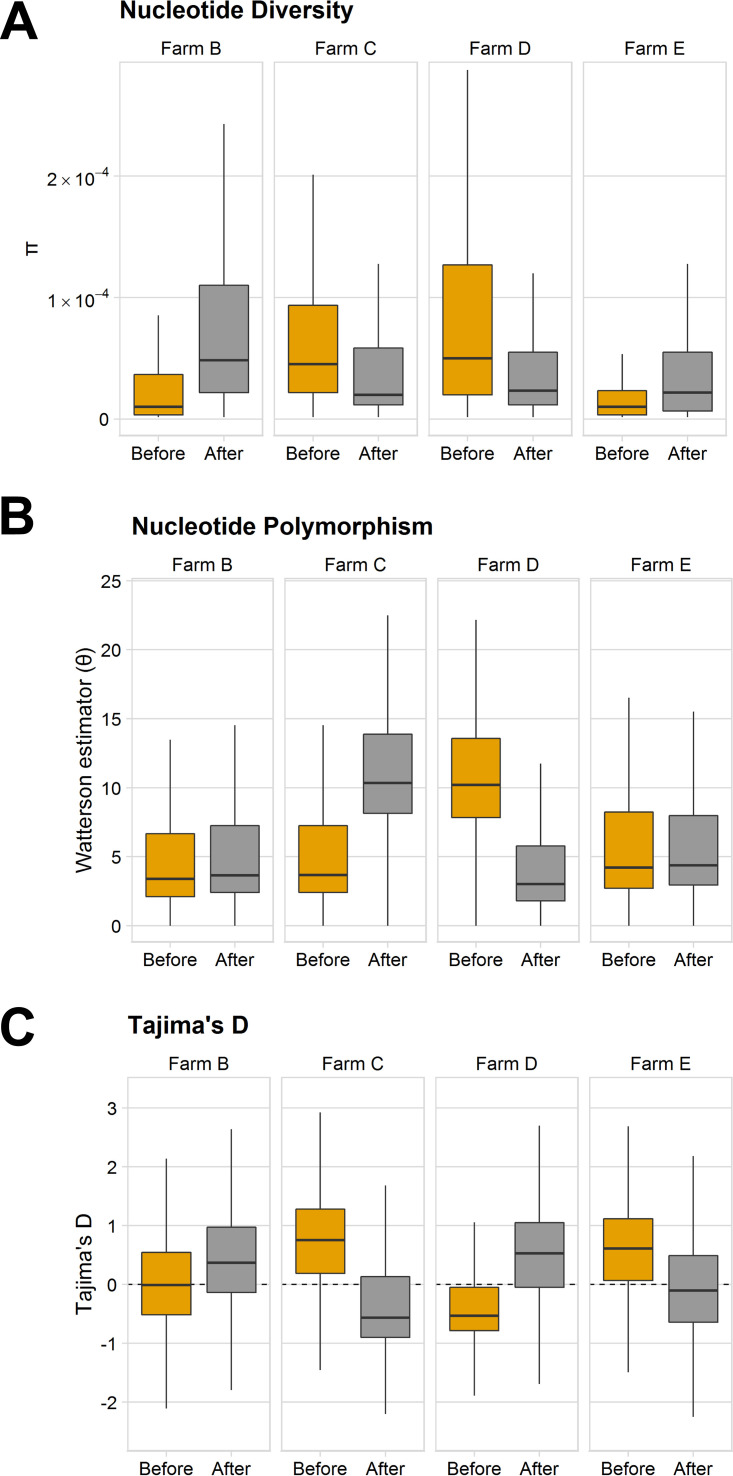
Genetic diversity among isolates from before and after the vegetative period and azole exposure. (A to C) Nucleotide diversity (π) (A), nucleotide polymorphism (Watterson estimator, or θ) (B), and Tajima’s D (C) along 5-kb windows with a 500-bp step size before and after the vegetative period and azole exposure. *n* = 8 isolates per farm and time point, 64 in total.

Given the observed reduction in overall A. fumigatus abundance after azole treatment, we examined the populations for changes in genetic diversity and evidence of selective sweeps in A. fumigatus field populations. Using 5-kb sliding windows with a 500-bp step size, nucleotide diversity (*π*) was calculated for the individual farms before and after azole treatment. No clear trend was seen regarding changes in nucleotide diversity between the time points. The isolates from farms B and E showed increased diversity following azole application, while nucleotide diversity decreased on farms C and D (Fig. 5A). We also calculated the number of segregating sites (*θ*) for the same 5-kb windows and observed the same lack of consensus. Farms B and C showed similar values of *θ* before compared to after azole application, while farm D showed a dramatic decrease in *θ* following azole application (Fig. 5B). AMOVA on isolates from before and after the vegetative period and azole exposure did not indicate any significant molecular variation between the time periods, with the majority of the variation being between and within samples (Table 4). Finally, we measured Tajima’s D to test neutrality along 5-kb windows, where negative values indicate less variation than expected and are indicative of a selective sweep. Positive values denote a population that is more heterogenous than would be expected and suggest either a sudden population contraction or balancing selection. Overall, the bulk of the Tamija’s D values were close to neutral, and there was no clear trend between farms, indicating that there was no genomic signature of a population bottleneck or selective sweep following azole exposure (Fig. 5C). The median Tajima’s D was roughly zero for farm B and increased slightly to 0.37 following azole exposure prior to azole application, and the same direction shift was seen for farm D, but the starting Tajima’s D was negative at the time point before azole exposure (−0.53 to 0.53) (Fig. 5C). Conversely, the median Tajima’s D for farms C and E shifted from positive to negative after the growing season and azole exposure (0.75 to −0.57 for field C and 0.61 to −0.10 for field E). Taken together, these results indicate that despite the reduction in abundance of A. fumigatus on agricultural fields following azole application, we were unable to detect any marked changes at the population level.

**TABLE 4:** Analysis of molecular variance between isolates collected before and after azole exposure^*a*^

Farm	Total variation	Variation within samples (%)	*P* value	Variation between samples (%)	*P* value	Variation between time periods (%)	*P* value
B	106,712.9	103,295.3 (96.8)	<0.001	3,729.5 (3.5)	<0.001	−311.9 (−0.3)	0.617
C	48,438.0	45,393.1 (93.7)	<0.001	3,341.7 (6.9)	<0.001	−296.8 (−0.6)	0.593
D	85,724.6	80,522.3 (93.9)	<0.001	5,592 (6.5)	<0.001	−389.7 (−0.5)	0.564
E	59,366.5	55,941.4 (94.2)	<0.001	3,001.2 (5.1)	<0.001	423.9 (0.7)	0.149

a *n* = 16 isolates per farm field (eight pre-azole and eight post-azole).

## DISCUSSION

The use of azole fungicides for plant protection has been previously suggested as a driver of clinical resistance in the environmental saprobe and human pathogen A. fumigatus, as well as the emergence of new fungal pathogens, such as Candida auris (11, 26, 27). However, direct evidence linking azole use in agriculture and clinical resistance is missing. Additionally, delineation of specific roles for the use of azoles in crops versus ornamental plants, such as flower bulbs, has not been defined and is important to make, as limiting azole fungicide use would significantly impact disease control and yield in many crops. In this study, we provide the first systematic investigation of the impact of fungicide use on the ecology and azole resistance status of a human pathogen, A. fumigatus. Through analysis of 2,875 soil samples over a 3-year period in central Germany, we found an overall low incidence of A. fumigatus isolates that would be considered clinically resistant (1 to 3%). However, we observed a modest, but consistent, decrease in azole susceptibility following the growing season and azole exposure, as well as a more variable reduction in fungal abundance following fungicide application. Interestingly, this change in susceptibility was transient and reset by the following spring. We also assessed the influence of fungicide application on A. fumigatus population dynamics by WGS and were unable to find a clear impact on the population structure or genetic diversity.

Despite sampling on fields that were actively treated with azole fungicides and in regions of Germany with above average fungicide exposure, only 1 to 3% of A. fumigatus isolates collected were resistant to medical azoles. This incidence is in agreement with clinically reported frequencies in Germany and other European countries, where the rate of triazole resistance ranged from 0.6% to 4.2% (3.2% in Germany) (28, 29). The environmental resistance rates reported for Europe, however, have been much broader. Some studies have found environmental resistance frequencies approaching 20%, while others have reported virtually no incidence of resistant environmental isolates (30–35). Part of these differences could be attributed to differences in methodology, such as the use of azole selection during the isolation procedure or inclusion of potentially clonal isolates from a given sample to skew the data. In the current study, we avoided azole selection during the isolation procedure and allowed only two isolates per soil sample to avoid potentially clonal isolates that influence the results. Another potentially contributing factor could be that while they are all technically “environmental” isolates, rural or agricultural settings could be a completely different niche than an urban flower garden, a supposition supported by a recent study where rural areas in the United Kingdom had much lower resistance rates (1.1%) than urban locations (13.8%) (34).

Another important and novel finding from this study is that isolates from after the vegetative period and azole exposure consistently showed decreased azole susceptibilities to difenoconazole and tebuconazole as well as a subtle MIC shift to both agricultural and medical azoles. However, one shortcoming to our study is that we did not collect isolates from organic fields at a matching time point for susceptibility testing, so we cannot exclude that the changes observed in azole susceptibility are not also influenced by seasonal changes. The observation that changes in susceptibility are transient and reset in the period between the end of the growth period and the following spring on the two fields is intriguing and worthy of further study. This transformation could either be the result of a naive population coming in via aerosol dispersal or a consequence of isolates acquiring unstable, epigenetic-mediated resistance, a phenomenon previously observed in the environmental saprobe and human pathogen Mucor circinelloides as well as the plant-pathogenic fungus *Monilinia fructicola* following azole exposure (36, 37). Either scenario would be in agreement with our finding that fungicide application does not alter the population structure or genetic diversity of A. fumigatus in agricultural fields.

We observed a wide range in the number of CFU per gram of soil within and between farms during annual spring sampling. Our mean number of A. fumigatus CFU per gram of soil was in line with what was reported recently for abundance in wheat grain, maize silage, and fruit waste (38). However, this abundance is several magnitudes lower than that reported for A. fumigatus in flower bulb waste and green material waste in this same study, where it was not uncommon to isolate 10^4^ CFU/g. We demonstrated a reduction in the A. fumigatus population size on most fields sampled in 2017 following the vegetative period and fungicide application. However, this finding was not strongly observed in 2018, indicating that other environmental factors also influence the abundance of A. fumigatus in agricultural soil. One example of such a potential factor is that Germany experienced extreme heat and drought during the 2018 growing season; in fact, one field had to be removed from analysis this year because it caught fire during the vegetative period.

Our study also provides the first WGS-based study focused on A. fumigatus in its natural niche, the environment. Previous studies have primarily concentrated on clinical isolates, with particular priority given to resistant strains (25, 39). Even while sampling within the same field, we found a large degree of genetic diversity, where the majority of diversity came from within samples. We also did not observe a defined population structure or separation between farms or regions. The degree to which this environmental diversity is recapitulated in clinical isolates, and whether there are enrichments for particular subgroups in the transition from environment to clinic, is an interesting question for further study.

Given our low observed resistance rate among agricultural isolates and the lack of discernible impact on the population structure and genomic diversity of A. fumigatus following fungicide application, our study does not find evidence that azole fungicide use in crop agriculture significantly contributes to resistance in A. fumigatus. Azoles should not necessarily be removed from use in this context due to their crucial role in global food production. Instead, our field study provides empirical support for the model that azole resistance in A. fumigatus is being driven not by the use of these compounds for crop agriculture but in settings such as the cultivation of flowers or ornamental plants as well as the storage of green waste. Both of these settings have been identified as hot spots for resistance development, owing to their higher overall fungal colony counts and higher fungicide concentrations (38, 40–42). Unfortunately, due to massive aerial dispersal of A. fumigatus conidia, the use of azoles in any hot spot can lead to the worldwide distribution of resistant strains.

## MATERIALS AND METHODS

### Site selection and soil sampling.

During 2016 to 2018, soil sampling was conducted on agricultural sites in the federal states of Thuringia, Saxony-Anhalt, and Saxony, with the approval of the land owner and/or relevant ministries. The majority of fields sampled were growing cereals such as wheat or barley, but some apple orchards were sampled as well (see Table S1 in the supplemental material for full details). Farms were arbitrarily assigned an alphabetic identifier (A to L) and specific fields a numeric identifier, as described in Text S1. Due to crop rotation, the same field could not be surveyed over subsequent years, with the exception of the apple orchards. Soil samples were collected at the beginning of the vegetation period on the conventional and organic cultivated sites, and after azole application an additional sampling was carried out on the conventional sites. In general, 50 soil samples per site and type of farming (conventional or organic) were collected, with a total of approximately 1,000 soil samples per year. Soil samples were selected to best cover the field with a minimum distance of 1 m between samples. For each sample, the top layer of soil was collected by a metal spatula into a sterile sample cup and refrigerated until processing.

10.1128/mBio.02213-20.9 TEXT S1 Supplemental materials and methods used in this study. Download Text S1, DOCX file, 0.02 MB. Copyright © 2020 Barber et al. 2020 Barber et al. This content is distributed under the terms of the Creative Commons Attribution 4.0 International license.

### Soil processing and isolation of A. fumigatus.

Three grams of soil from each sample cup was weighed out and resuspended in 8 ml 0.2 M NaCl containing 1% Tween 20. Samples were vortexed vigorously and then left to settle until a phase separation became apparent. Two milliliters of the upper phase was transferred to a new tube for plating onto Sabouraud glucose agar (SGA) containing 50 μg/ml chloramphenicol (Sigma-Aldrich, Taufkirchen, Germany). Of this 2 ml, 150 μl was plated onto one plate and the remaining volume was plated onto a second plate to adjust for variable fungal concentrations in samples. Plates were then incubated at 50°C for 5 days to select for A. fumigatus, which is unique among *Aspergillus* spp. in its ability to grow at this temperature. On day 5, the incubator temperature was reduced to 42°C to allow for sporulation, and plates were grown for another 2 days. The number of A. fumigatus colonies was counted, and up to two colonies per soil sample were transferred to new plates for isolation.

### Antifungal susceptibility testing.

Quick screening of susceptibility to itraconazole, voriconazole, and posaconazole was assessed using the agar-based VIPcheck assay (Mediaproducts BV, Groningen, Netherlands) by following the manufacturer’s directions. For testing, isolates were grown on SGA for 2 to 4 days at 37°C. Plates were then swabbed with a damp, sterile cotton swab to prepare a conidial suspension of 0.5 to 2 McFarland. Twenty-five microliters of this suspension was then plated onto the 4 wells of the VIPcheck plate containing 4 mg/liter itraconazole, 2 mg/liter voriconazole, or 0.5 mg/liter posaconazole or a control well containing no drug. To assess susceptibility to agricultural azoles for isolates collected during 2017 and 2018, RPMI plus 2% glucose agar plates containing either 1 mg/liter difenoconazole or 2 mg/liter tebuconazole was prepared as described in reference 43. Resistance was defined as significant inhibition of germination and hyphal growth compared to the no drug control. Isolates that showed resistance to any of the medical azoles in the VIPcheck assay were subject to broth microdilution following EUCAST methodology (protocol E.DEF 9.3). ECOFFs were calculated using the ECOFFinder program available from EUCAST.

### DNA extraction.

For WGS and PCR-based amplification, isolates were grown shaking in SG broth at 37°C, and genomic DNA was isolated using the Quick-DNA fungal/bacterial miniprep kit (Zymo Research, Irvine, CA) according to the manufacturer’s suggested protocol.

### *cyp51a* genotyping.

The *cyp51a* coding sequence and upstream region containing the tandem repeat was amplified using the primers described in Table S5. Cleaned-up PCR products were sequenced and the *cyp51a* genotype determined using FunResDB (https://elbe.hki-jena.de/FunResDb/index.php).

### Genome sequencing, quality assessment, and alignment.

Library preparation and 2 × 150-bp paired-end sequencing were performed on a NextSeq 500 v2 by LGC Genomics (Berlin, Germany) by following the manufacturer’s recommended protocols. Sequence data quality control and filtering were performed using an in-house script and FastQC (v0.11.5). Quality reads were mapped to the A. fumigatus Af293 reference genome (version 2015-09-27; retrieved from FungiDB [44]) using BWA-MEM (version 0.7.8-r779-dirty) (45). PCR duplicates were marked using MarkDuplicate from Picard version 2.18.25 embedded in the Genome Analysis Toolkit (GATK; version 4.1.0.0). All WGS samples included for analysis possessed greater than 10-fold genome coverage after mapping, and more than 90% of reads mapped to the reference genome.

### Variant identification and SNV-based phylogeny.

Short variants, including single-nucleotide variants (SNVs) and short insertions and deletions (InDels), were detected using GATK Haplotype Caller by following the recommended best practices for single calling (46). Copy number variants (CNVs) were identified using Control-FREEC (47). For the phylogenetic analysis, nucleotide consensus sequences were extracted from vcf files using VCFtools (48), and an in-house script was used to translate nucleotides to protein-coding sequences. Multiple-sequence alignment was performed using MUSCLE v3.8.31 (49) with 7,771 conserved core genes and an approximately maximum likelihood phylogeny constructed using FastTree2 (version 2.1.10) (50). The Interactive Tree of Life (iTOL) v4 was used for visualization (51).

### Genetic diversity analyses.

Analysis of molecular variance (AMOVA) was determined using the R package ade4 (nrep = 999). Nucleotide diversity (π) was calculated by VCFtools (version 0.1.6) using 5-kbp windows with a step size of 500 bp. Nucleotide polymorphism (θ) and Tajima’s D were calculated using ANGSD (52) with a window size of 5 kbp and a step size of 500 bp. Weighted Weir and Cockerham’s F_ST_ values were calculated using VCFtools, while V_ST_ values were calculated as in reference 53. A Mantel test correlating geographic distance matrices with pairwise F_ST_ matrices was performed using the R package ade4 (nrep = 9999).

### Estimation of fungicide treatment areas and rates in Germany.

The fraction of each district theoretically treated with fungicides was calculated using publicly reported data from 2016, available from the German Statistical Offices (https://statistikportal.de), using the following equation:

$$\frac{({\text{Ha}}_{\text{Ackerland}}+{\text{Ha}}_{\text{Dauerkulturen}})\times {\text{Fraction}}_{\text{Conventional}}}{{\text{Ha}}_{\text{Total}}}$$

The total area per district was obtained from Table 33111-01-02-4, ground area by actual use. The sum of arable farmland and permeant crop areas was calculated for each administrative district using Table 41141-01-01-4, farms and their agricultural use area by crop type. To accommodate that some percentage of this area is cultivated under organic agriculture methods, the hectares of cropland were then multiplied by the fraction of nonorganic agriculture for the federal state in which the district is located to estimate the number of hectares potentially treated with fungicides (as calculated using data on farms, agricultural areas, and workers; accessed on 1 November 2019 from https://www.statistikportal.de/node/254). Unfortunately, no data were available on the breakdown of agricultural methods at the district level to allow for more exact estimation. Finally, this value of estimated treated area per district was divided by the total area of the district for visualization as a choropleth map.

### Box and whisker plots.

Box and whisker plots presented in this paper are in the style of Tukey, where the boldface line indicates the 50th percentile and the hinges represent the 25th and 75th percentiles. The lower whisker extends from the lower hinge to the lowest datum within a 1.5 interquartile range (IQR), while the upper whisker represents the highest datum still within 1.5 IQR. Outliers are marked with points.

### Data and isolate availability.

Isolates generated within this study were submitted to and are publicly available in the Jena Microbial Resource Collection. Raw FASTQ files were uploaded to the NCBI Sequence Read Archive and are publicly available under BioProject number PRJNA595552.
