# Supplementary material for: Effects of Agricultural Fungicide Use on Aspergillus fumigatus Abundance, Antifungal Susceptibility, and Population Structure
Source: mBio. 2020 Nov 24;11(6):e02213-20. doi: 10.1128/mBio.02213-20 (PMC7701986; doi:10.1128/mBio.02213-20)
Supplement: TABLE S1 [file mBio.02213-20-st001.docx]

**Supplemental Table 1**. Summary of the agricultural sites surveyed 2016- 2018.

| Farm | Type of Agriculture | Crop | Soil samples (n) | | | *A. fumigatus* isolates (n) | | |
| --- | --- | --- | --- | --- | --- | --- | --- | --- |
|  |  |  | 2016 | 2017 | 2018 | 2016 | 2017 | 2018 |
| A | Conventional and organic | Cereal | 150 | 150 | 150 | 232 | 94 | 81 |
| B | Conventional | Cereal | 100 | 100 | 100 | 114 | 266 | 335 |
| C | Conventional | Cereal | 100 | 100 | 100 | 29 | 123 | 37 |
| D | Conventional | Cereal | 100 | 100 | 100 | 65 | 228 | 287 |
| E | Conventional | Cereal | 100 | 100 | 100 | 61 | 131 | 165 |
| F | Organic | Cereal | 50 | 50 | 25 | 73 | 87 | 15 |
| G | Organic | Cereal | 50 | 50 | 50 | 29 | 87 | 13 |
| H | Conventional and organic | Apple | 100 | 150 | 150 | 158 | 301 | 421 |
| K | Organic | Cereal | 50 | 50 | 50 | 45 | 26 | 6 |
| L | Conventional and organic | Apple | 100 | 150 | 150 | 91 | 184 | 162 |
| Total | | | 900 | 1000 | 975 | 897 | 1527 | 1522 |
