# Supplementary material for: Effects of Agricultural Fungicide Use on Aspergillus fumigatus Abundance, Antifungal Susceptibility, and Population Structure
Source: mBio. 2020 Nov 24;11(6):e02213-20. doi: 10.1128/mBio.02213-20 (PMC7701986; doi:10.1128/mBio.02213-20)
Supplement: TABLE S2 [file mBio.02213-20-st002.docx]

**Supplemental Table 2**. Resistance summary of the fields sampled during the spring of 2017 (A) and 2018 (B).

**(A)**

|  | | | | Proportion of isolates that grow  at the concentration indicated | | | | |
| --- | --- | --- | --- | --- | --- | --- | --- | --- |
| Type of agriculture | Field | Crop | n tested | DIF  (1 mg/L) | TEB  (2 mg/L) | ITR  (4 mg/L) | VOR  (2 mg/L) | POS  (0.5 mg/L) |
| Conventional | A-1-17 | Cereal | 20 | 0.15 | 0.00 | 0.00 | 0.00 | 0.00 |
|  | B-1-17 | Cereal | 20 | 0.25 | 0.05 | 0.00 | 0.00 | 0.00 |
|  | C-1-17 | Cereal | 20 | 0.10 | 0.00 | 0.00 | 0.00 | 0.00 |
|  | D-1-17 | Cereal | 20 | 0.25 | 0.00 | 0.00 | 0.00 | 0.00 |
|  | D-2-17 | Cereal | 20 | 0.40 | 0.05 | 0.00 | 0.00 | 0.00 |
|  | E-1-17 | Cereal | 20 | 0.15 | 0.05 | 0.00 | 0.00 | 0.00 |
|  | H-1-17 | Apple | 20 | 0.15 | 0.05 | 0.00 | 0.00 | 0.00 |
|  | L-1-17 | Apple | 20 | 0.15 | 0.00 | 0.00 | 0.00 | 0.00 |
|  | L-4-17 | Apple | 20 | 0.55 | 0.00 | 0.00 | 0.00 | 0.00 |
| Organic | A-2-17 | Cereal | 20 | 0.35 | 0.15 | 0.05 | 0.00 | 0.00 |
|  | F-1-17 | Cereal | 20 | 0.25 | 0.00 | 0.00 | 0.00 | 0.00 |
|  | G-1-17 | Cereal | 20 | 0.25 | 0.10 | 0.05 | 0.00 | 0.00 |
|  | G-2-17 | Cereal | 20 | 0.15 | 0.00 | 0.00 | 0.00 | 0.00 |
|  | K-1-17 | Cereal | 20 | 0.25 | 0.00 | 0.00 | 0.00 | 0.00 |
|  | H-2-17 | Apple | 20 | 0.25 | 0.00 | 0.00 | 0.00 | 0.00 |
|  | L-2-17 | Apple | 20 | 0.10 | 0.05 | 0.00 | 0.00 | 0.00 |
|  | L-3-17 | Apple | 20 | 0.35 | 0.25 | 0.00 | 0.00 | 0.00 |

**(B)**

|  | | | | Proportion of isolates that grow  at the concentration indicated | | | | |
| --- | --- | --- | --- | --- | --- | --- | --- | --- |
| Type of agriculture | Field | Crop | n tested | DIF  (1 mg/L) | TEB  (2 mg/L) | ITR  (4 mg/L) | VOR  (2 mg/L) | POS  (0.5 mg/L) |
| Conventional | A-3-18 | Cereal | 11 | 0.27 | 0.00 | 0.00 | 0.00 | 0.00 |
|  | B-2-18 | Cereal | 20 | 0.10 | 0.05 | 0.00 | 0.00 | 0.00 |
|  | C-2-18 | Cereal | 11 | 0.09 | 0.00 | 0.00 | 0.00 | 0.00 |
|  | D-3-18 | Cereal | 20 | 0.10 | 0.05 | 0.00 | 0.00 | 0.00 |
|  | E-2-18 | Cereal | 20 | 0.10 | 0.10 | 0.00 | 0.00 | 0.00 |
|  | H-1-18 | Apple | 20 | 0.20 | 0.10 | 0.00 | 0.00 | 0.00 |
|  | L-1-18 | Apple | 18 | 0.11 | 0.00 | 0.00 | 0.00 | 0.00 |
|  | L-4-18 | Apple | 12 | 0.17 | 0.00 | 0.00 | 0.00 | 0.00 |
| Organic | A-4-18 | Cereal | 20 | 0.40 | 0.20 | 0.00 | 0.00 | 0.00 |
|  | F-2-18 | Cereal | 10 | 0.40 | 0.00 | 0.00 | 0.00 | 0.00 |
|  | G-3-18 | Cereal | 12 | 0.50 | 0.00 | 0.00 | 0.00 | 0.00 |
|  | K-2-18 | Cereal | 5 | 0.00 | 0.00 | 0.00 | 0.00 | 0.00 |
|  | H-2-18 | Apple | 20 | 0.20 | 0.05 | 0.00 | 0.00 | 0.00 |
|  | L-2-18 | Apple | 6 | 0.17 | 0.00 | 0.00 | 0.00 | 0.00 |
|  | L-3-18 | Apple | 8 | 0.38 | 0.00 | 0.00 | 0.00 | 0.00 |
