# Supplementary material for: Effects of Agricultural Fungicide Use on Aspergillus fumigatus Abundance, Antifungal Susceptibility, and Population Structure
Source: mBio. 2020 Nov 24;11(6):e02213-20. doi: 10.1128/mBio.02213-20 (PMC7701986; doi:10.1128/mBio.02213-20)
Supplement: TABLE S3 [file mBio.02213-20-st003.docx]

**Supplemental Table 3**. Resistance summary of the fields sampled before and after the vegetative period and azole application in 2017 (A) and 2018 (B).

**(A)**

|  | | | | Proportion of isolates that grow  at the concentration indicated | | | | |
| --- | --- | --- | --- | --- | --- | --- | --- | --- |
| Field | Crop | Time Period | n tested | DIF  (1 mg/L) | TEB  (2 mg/L) | ITR  (4 mg/L) | VOR  (2 mg/L) | POS  (0.5 mg/L) |
| B-1-17 | Cereal | Before | 20 | 0.25 | 0.05 | 0.00 | 0.00 | 0.00 |
|  |  | After | 20 | 0.50 | 0.15 | 0.00 | 0.00 | 0.00 |
| C-1-17 | Cereal | Before | 20 | 0.10 | 0.00 | 0.00 | 0.00 | 0.00 |
|  |  | After | 20 | 0.45 | 0.15 | 0.10 | 0.00 | 0.00 |
| D-1-17 | Cereal | Before | 20 | 0.25 | 0.00 | 0.00 | 0.00 | 0.00 |
|  |  | After | 20 | 0.50 | 0.25 | 0.00 | 0.00 | 0.00 |
| D-2-17 | Cereal | Before | 20 | 0.40 | 0.05 | 0.00 | 0.00 | 0.00 |
|  |  | After | 20 | 0.35 | 0.05 | 0.00 | 0.00 | 0.00 |
| E-1-17 | Cereal | Before | 20 | 0.15 | 0.05 | 0.00 | 0.00 | 0.00 |
|  |  | After | 15 | 0.47 | 0.07 | 0.00 | 0.00 | 0.00 |
| H-1-17 | Apple | Before | 20 | 0.15 | 0.05 | 0.00 | 0.00 | 0.00 |
|  |  | After | 20 | 0.55 | 0.20 | 0.00 | 0.00 | 0.00 |
| L-4-17 | Apple | Before | 20 | 0.55 | 0.00 | 0.00 | 0.00 | 0.00 |
|  |  | After | 20 | 0.50 | 0.10 | 0.00 | 0.00 | 0.00 |

**(B)**

|  | | | | Proportion of isolates that grow  at the concentration indicated | | | | |
| --- | --- | --- | --- | --- | --- | --- | --- | --- |
| Field | Crop | Time Period | n tested | DIF  (1 mg/L) | TEB  (2 mg/L) | ITR  (4 mg/L) | VOR  (2 mg/L) | POS  (0.5 mg/L) |
| A-3-18 | Cereal | Before | 11 | 0.27 | 0.00 | 0.00 | 0.00 | 0.00 |
|  |  | After | 11 | 0.27 | 0.18 | 0.00 | 0.00 | 0.00 |
| B-2-18 | Cereal | Before | 20 | 0.10 | 0.05 | 0.00 | 0.00 | 0.00 |
|  |  | After | 20 | 0.25 | 0.10 | 0.05 | 0.00 | 0.00 |
| C-2-18 | Cereal | Before | 11 | 0.09 | 0.00 | 0.00 | 0.00 | 0.00 |
|  |  | After | 16 | 0.25 | 0.06 | 0.00 | 0.00 | 0.00 |
| D-3-18 | Cereal | Before | 20 | 0.10 | 0.05 | 0.00 | 0.00 | 0.00 |
|  |  | After | 20 | 0.35 | 0.10 | 0.05 | 0.00 | 0.00 |
| H-1-18 | Apple | Before | 20 | 0.20 | 0.10 | 0.00 | 0.00 | 0.00 |
|  |  | After | 20 | 0.50 | 0.10 | 0.00 | 0.00 | 0.00 |
| L-1-18 | Apple | Before | 18 | 0.11 | 0.00 | 0.00 | 0.00 | 0.00 |
|  |  | After | 10 | 0.50 | 0.00 | 0.00 | 0.00 | 0.00 |
| L-4-18 | Apple | Before | 12 | 0.17 | 0.00 | 0.00 | 0.00 | 0.00 |
|  |  | After | 12 | 0.58 | 0.08 | 0.00 | 0.00 | 0.00 |
