# Supplementary material for: Effects of Agricultural Fungicide Use on Aspergillus fumigatus Abundance, Antifungal Susceptibility, and Population Structure
Source: mBio. 2020 Nov 24;11(6):e02213-20. doi: 10.1128/mBio.02213-20 (PMC7701986; doi:10.1128/mBio.02213-20)
Supplement: TABLE S4 [file mBio.02213-20-st004.docx]

**Supplemental Table 4**. MICs for medical and agricultural azoles in isolates displaying resistance to at least one medical azole.

| **Strain** | ***cyp51a* genotype**^+^ | **ITR** | **POS** | **VOR** | **DIF** | **TEB** | **AMB** |
| --- | --- | --- | --- | --- | --- | --- | --- |
| 2016-072 | TR34/L98H | >8 | 1 | 4 | >8 | >8 | 0.25 |
| 2016-262 | TR34/L98H | >8 | 1 | 4 | >8 | >8 | 0.25 |
| 2016-334 | TR34/L98H | >8 | 0.5 | 2 | 8 | >8 | 0.25 |
| 2016-375 | TR34/L98H | >8 | 0.5 | 4 | 4 | >8 | 0.5 |
| 2016-439 | TR34/L98H | >8 | 1 | 4 | >8 | >8 | 0.25 |
| 2016-698 | TR34/L98H | >8 | 1 | 4 | >8 | >8 | 0.25 |
| 2017-105 | TR34/L98H | >8 | 1 | 4 | >8 | >8 | 0.25 |
| 2017-402 | TR34/L98H | >8 | 1 | 2 | 8 | >8 | 0.25 |
| 2017-415 | TR34/L98H | >8 | 1 | 4 | 8 | >8 | 0.25 |
| 2016-106 | WT | >8 | 0.25 | 1 | 4 | 8 | 0.5 |
| 2017-153 | WT | >8 | 0.5 | 2 | 4 | 8 | 0.25 |
| 2016-684 | WT | 8 | 0.5 | 1 | 4 | 8 | 0.5 |
| 2016-313 | WT | 4 | 0.25 | 1 | 8 | >8 | 0.5 |
| 2016-675 | WT | 4 | 0.5 | 2 | 8 | 8 | 0.5 |
| 2016-369 | WT | 2 | 0.125 | 1 | 2 | 4 | 0.25 |
| 2018-214 | WT | 2 | 0.25 | 1 | 4 | 4 | 0.25 |
| 2018-272 | WT | 2 | 0.125 | 1 | 4 | 4 | 0.25 |

Abbreviations: amphotericin B = AMB; difenoconazole = DIF; itraconazole = ITR; posaconazole = POS; tebuconazole = TEB; voriconazole = VOR; WT = wild type.

^+^Relative to ATCC36607.
