# Supplementary material for: Effects of Agricultural Fungicide Use on Aspergillus fumigatus Abundance, Antifungal Susceptibility, and Population Structure
Source: mBio. 2020 Nov 24;11(6):e02213-20. doi: 10.1128/mBio.02213-20 (PMC7701986; doi:10.1128/mBio.02213-20)
Supplement: TABLE S5 [file mBio.02213-20-st005.docx]

**Supplemental Table 5**. Primers used for amplifying and sequencing *cyp51a*.

| **Primer** | **Sequence** | **Use** |
| --- | --- | --- |
| cyp51a_F | 5’-CGTAGCAAGGGAGAAGGAAA | Amplification and sequencing |
| cyp51a_R | 5’-CACCTATTCCGATCACACCA | Amplification and sequencing |
| cyp51a_internal | 5’-ATGTCAATGCGGAAGAGGTC | Sequencing |
